# Association between Sleep and Body Weight: A Panel Data Model Based on a Retrospective Longitudinal Cohort of Chinese Infants

**DOI:** 10.3390/ijerph14050458

**Published:** 2017-04-25

**Authors:** Tingting Sha, Yan Yan, Xiao Gao, Shiting Xiang, Guangyu Zeng, Shiping Liu, Qiong He

**Affiliations:** Department of Epidemiology and Medical Statistics, Xiangya School of Public Health, Central South University, Changsha 410078, Hunan, China; tingtingsha@csu.edu.cn (T.S.); 18670321975@163.com (X.G.); TC_konnext@126.com (S.X.); 15873124652@139.com (G.Z.); liuship92@163.com (S.L.); qqniuniu0525@163.com (Q.H.)

**Keywords:** sleep duration, body weight, panel data, fixed effects, obesity

## Abstract

The focus of this article is on sleep duration and sleep problems in infants and their association with body weight. A retrospective birth cohort of 519 infants was enrolled in a community-based study conducted in Changsha, China. Infant weight and other health-related information were collected during regular standard checkups at the Community Health Service Centers when infants were 1, 3, 6, 8, and 12 months old. The sleep duration and sleep problems of infants were assessed by maternal self-reports. Panel data model was used to evaluate the association of sleep duration and sleep problems with infant body weight. Significant relevance between self-reported sleep duration and weight of infants has been reported in the literature tested by the fixed effects model (*p* < 0.01). However, this study indicated that sleep problems of infants had no effect on their weight (*p* = 0.151), after adjusting feeding patterns and socioeconomic factors of their families. This paper argues that, as a potentially modifiable risk factor, infant sleep duration deserves more attention from their parents and families in order to prevent and control overweight or obesity in infants as well as reducing the incidence of obesity in adults.

## 1. Introduction

Childhood obesity has been one of the most serious public health challenges over the past decades [1]. This problem is affecting many low- and middle-income countries globally and steadily [2], particularly in Asian countries [3]. The incidence of childhood obesity has increased overwhelmingly. Over 42 million children under the age of five, are threatened by obesity or overweight universally [4]. Worse, it is worthy of our attention that the occurrence of fast-growing weight or even transiently rapid weight gain during infancy might contribute to overweight and obesity in childhood and adulthood [5,6,7,8,9,10]. Moreover, once the obesity occurs, it is difficult to be reversed through interventions [11]. Obesity or overweight, as well as their related risk factors, can be easily controlled and prevented. Therefore, prevention of infant obesity needs high priority.

Sleep deprivation as a possible contributor to obesity or overweight has received much attention in scientific literature in recent years [12]. A considerable amount of literature has indicated that short sleep duration may result in excess weight gain and obesity by causing hormonal and metabolic changes [13]. Furthermore, shorter sleep duration having an influence on weight from the genomic levels by increasing the expression of genetic risks for excess body weight has been proven by previous studies [14]. In addition, recent studies claim that frequent night waking or settling problems are related to a variety of health problems including obesity, metabolic syndromes, and growth retardation [15,16]. Based on this evidence, prevention and early control of short sleep duration and sleep problems might help in reducing the incidence of obesity and providing the intervention strategies to reduce the regional burden of disease.

Although most of research has proven a consistent association between sleep and body weight among adults [17,18,19], there are limited studies on the question of these relationships among infants [9,20]. A few studies showed that infants who sleep less than 12 h/d and who have frequent night waking were associated with higher body mass index and increased risks of being overweight at three years old [21,22], while another study showed no significant association between these factors [15]. Changes and risk factors of infant weight differ greatly from that of an adult’s. Compared with adults, weight changes more rapidly in infants and is influenced by growth hormones, feeding patterns, genetic factors, and many other factors [23]. Furthermore, the growing attention on the links between sleep and infant weight were affected by various limitations: (1) most existing studies were cross sectional studies which provide weak evidence to demonstrate causality; (2) The sample size of the study conducted by Tikotzky et al. [22] was only 96 which was not large enough for testing the association between infant sleep and weight; (3) In addition, almost all of the previous studies corroborated the sleep-weight association through traditional statistical methods, such as partial correlations, stepwise regression analysis, and so on. These traditional methods can only control observed confounding factors, however, they cannot adjust the unobserved confounders such as genes, which might draw fallacious conclusions. Given the limitations of current studies, the association between sleep and body weight among infants remains unclear and requires further epidemiological studies to identify.

To our knowledge, a longitudinal study is better in demonstrating causality association of infant sleep with weight than cross sectional study. Compared with the traditional statistical analysis, the panel data model can control both observed and unobserved confounding factors within individuals [24,25] and is more suitable for the longitudinal data analysis by connecting individual experience and behaviors at different time points [25,26,27,28]. In this study, we aim to reconsider sleep duration and sleep problems in infants and their association with body weight by using the panel data model analysis based on a retrospective longitudinal cohort of Chinese infants.

## 2. Materials and Methods

### 2.1. Subjects and Sampling

This study is a retrospective longitudinal cohort of Chinese infants, and it aims to provide an insight into the weight changes of infants and to examine the association between infant sleep and infant weight. The Community Health Service Centers of Sifangping, Dongfenglu, Xinhe streets of Kaifu District in Changsha, China were selected as the investigation sites. Random sampling method was used to select the infants who were born in these Community Health Service Centers during 2013. Data were collected at five time points: 1, 3, 6, 8, and 12 months old in infancy. Eligibility requirements of subjects specified: (1) mothers and their infants who live in Changsha and have completed health records at any of the Community Health Service Centers; (2) mothers who have no cardiovascular diseases and other organic diseases; (3) infants who are not twins, multiple fetuses and without congenital diseases. Finally, 575 infants were recruited in this study at the baseline survey. After excluding failed follow-ups and incomplete records such as missing key information like infant weight (*n* = 56), 519 respondents who completed all the follow-ups as well as meeting the inclusion criteria were included.

### 2.2. Data Collection

#### 2.2.1. Measurement of Infant Weight

To increase the reliability of measures, each infant weight was tested twice with an electronic portable scale with a precision of 0.1 kg at five time points: 1, 3, 6, 8, and 12 months old in infancy. Data were collected by the doctors of Community Health Service Centers at regular checkups during the year of 2013. Infant weight was calculated by subtracting the clothes weight from the average of two measurements. Birth weight was collected from the infant’s maternity card. Weight-for-age Z-score was calculated by using the principles of World Health Organization Growth Standard (2006). Infant’s nutritional status was divided into four categories namely malnutrition, normal weight, overweight, and obesity, according to weight-for-age Z-score [29].

#### 2.2.2. Measurement of Infant Sleep

Data regarding infant sleep were obtained by maternal reports of infant behaviors at 1–12 months of age. Throughout this paper infant sleep was assessed using maternal reports of infant sleep duration and infant sleep problems. Infant sleep duration in the last week was acquired by asking mothers question like: “What is the average total time slept during the day and at night of your baby in a 24-h period?” Infant sleep problems was defined as waking up three times or even more per night, or parents reporting “severe” disturbance proposed by Zuckerman [30]. In this study, it was assessed by asking mothers to respond “yes” or “no” to the following question: “Does your baby has sleep problems such as night waking or settling problems at night recently?”

#### 2.2.3. Factors of Families and Infants

The self-made family and infant questionnaire was used to collect two aspects of information by face-to-face interviews, including infant and family socioeconomic factors and infant feeding patterns. Maternal age, educational level, marital status, infant gender, and per capita income of households were first collected in the demographic information questionnaire. Infant feeding patterns, time of outdoor activities, taking Vitamin D, and other health information were collected by the interviewers when infants were at 1, 3, 6, 8, and 12 months of age. While a variety of definitions of the exclusive breastfeeding have been suggested, this study uses the latest definition proposed by the World Health Organization and the United Nations International Children’s Emergency Fund on the 55th World Health Assembly in May 2001, that is, 0 to 6 months old infants shall not accept any other food, drink, and even water besides breast milk. Artificial feeding referred to mothers who did not have breast milk and needed milk substitutes to feed their infants. Mixing feeding was defined as the insufficient supply of a mother’s breast milk which requires the compensation of the milk substitutes to meet the needs of their infant’s growth.

### 2.3. Data Analysis

The superiority of the panel data model is that it can increase the estimator precision by increasing the number of observations and obtain more dynamic information than a single cross sectional data [28], which can reflect the optimal validity of data [27]. Furthermore, the fixed effects model can eliminate the influence of individual-variant but time-invariant unobserved confounders in the study [31]. There are three kinds of panel data models namely, pooled effects model, fixed effects model, and random effects model. The basic linear panel models can be described through suitable restrictions of the following general model:

(1) $$y_{it}=\alpha_{it}+\mu_{it}+X_{it}\beta_{it},$$

where *i* = 1…N refers to the individual index, *t* = 1. T is the time index and $\mu_{it}$ refers to a random disturbance term of mean 0. $y_{it}$ refers to an explained variable, $\alpha_{it}$ refers to intercept, $X_{it}$ refers to an explanatory variable,$\;\beta_{it}$ represents the coefficient of $X_{it}$.

When α_it_ = α, for all *i*, *t* and $\beta_{it}$ = β, for all i, t, the model turns into a standard linear model pooled.

(2) $$y_{it}=\alpha +\mu_{it}+X_{it}\beta ,$$

When α_it_ = α_i_, for all *t*, the resulting model is called the fixed effects model,

(3) $$y_{it}=\alpha_{i}+\mu_{it}+X_{it}\beta ,$$

If the individual-specific component $\mu_{it}$ is uncorrelated with the regressors, the situation is usually termed random effects model.

The data was checked manually for completeness and input via EpiData version 3.1 (EpiData Association, Odense, Denmark) by two investigators. Nutritional status such as malnutrition rates were compared between different months old infants using the χ^2^ test. Change trends of socioeconomic factors of infant’s families among different time points were examined by using generalized estimating equations via statistical analysis software PASW (Predictive Analytics Software) statistics version 18.0 (SPSS, Chicago, IL, USA). Using the plm package of R software version 3.3.1 (Foundation for Statistical Computing, Vienna, Austria) to investigate the relationship between infant sleep and infant body weight based on the panel data model. Significance level was set to *p* < 0.05, *p* was bilateral and was used for all analyses. The different follow-up months were seen as observation time points and each infant was viewed as a cross section. The outcome variable in the study represented the infant weight in kg at each time point. The potential influencing factors of infant weight, such as sleep duration and feeding patterns, were viewed as the explanatory variables in the panel data model. This study collected 5 × 519 data points in total. At first, the data was fitted with the pooled effects model, fixed effects model and random effects model respectively. Then, using the F test to choose between fixed and pooled effects specifications, and comparing the two estimators under the null hypothesis of no significant difference: if this is rejected, then the more efficient fixed effects estimator is chosen. The selection for fixed and random effects specifications is based on Hausman-type test, if this is rejected, a fixed effects model is chosen [32]. At last, according to the results, reasonable panel data model was chosen to elaborate the real association between infant sleep and their body weight after controlling potential family socioeconomic factors.

### 2.4. Ethical Approval

This study was approved by the Ethics Committee (EC) of clinical pharmacology institute of Central South University (CTXY-130041-3-2). Participants were told that the information collected would be kept strictly confidential by the investigators. The parents who met the inclusion criteria and wrote the informed consent were involved in this study.

## 3. Results

### 3.1. General Description of Infants and Their Mothers

Table 1 summarizes the characteristics of the subjects. This study consecutively enrolled 519 cases with complete information, including 268 males and 251 females. The average infant’s birth weight and height were (3.31 ± 0.43) kg and (49.87 ± 1.08) cm, respectively. The average gestational age of infants was (39.06 ± 1.76) weeks.

**Figure 1:**
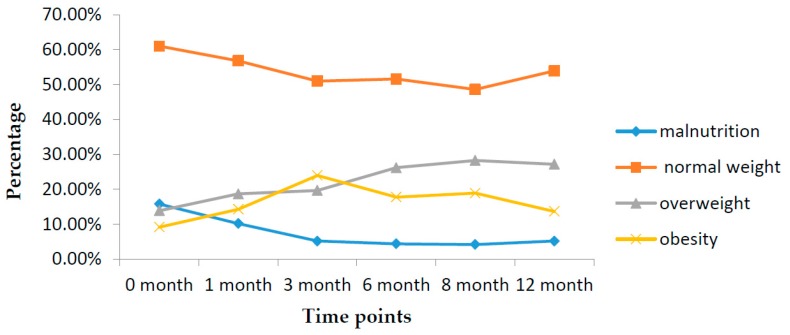
Prevalence of malnutrition, normal weight, overweight, and obesity infants aged 0–12 months old in Changsha, China in 2013.

Table 2 shows the results of χ^2^ test for the nutritional status such as malnutrition rates of different months old infants. Significant differences of the nutritional status were found and summarized in Table 2. In general, decreasing trends across the five time points were observed in the prevalence of malnutrition and normal weight from 15.8% to 5.2% and 61.0% to 53.9%, respectively. By contrast, it was found that the proportion of infant overweight increased with age from 13.9% to 27.2% and peaked at eight months of age, reaching up to 28.3%. Obesity occurred mostly at three months old, accounting for 24.0% (Figure 1).

Table 3 shows the generalized estimating equations results based on the individual level for the differences of infant and family socioeconomic factors across the five time points. Infant month age was viewed as the independent variable while weight, number of breast-feedings per day, sleep problems, and other variables were viewed as the dependent variables in the generalized estimating equations. Infant sleep duration declined from 17.78 h to 12.29 h during infancy (*p* < 0.05). This study did not find out any evidence that infant sleep problems had significant differences across different time points (*p* = 0.344). Change trends of infant weight, number of formula per day, and the time of outdoor activities increased continuously with age (*p* < 0.05). Similarly, the increased proportion of infant formula feeding and complimentary feeding were observed across the five time points (*p* < 0.05). The number of breast-feedings per day and breast-feeding rate declined sharply during infancy (*p* < 0.05).

The results for the correlation of infant sleep duration and sleep problems with infant weight across different time points are presented in Table 4. Significant negative correlations between sleep duration and infants weight have been reported in the study when infants were at 1, 3, 6 months old, respectively (*p* < 0.05). However, such relevance is not statistically significant after infants reached 8 months old. This study did not find out any evidence that infants sleep problems had a significant difference on their body weight during infancy.

### 3.2. The Panel Data Analysis

**Figure 2:**
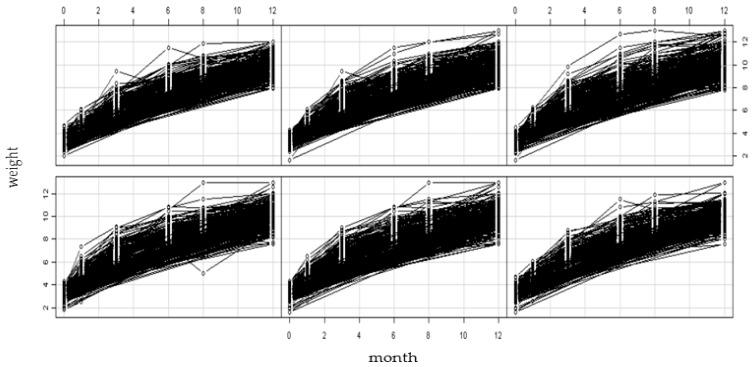
The weight growth tracks for 519 infants aged from 0 to 12 months old were represent based on panel data via R software. In order to look clearly, we divided the weight growth tracks into six equal parts in the picture according to their sequential panel ID. Each curve was corresponding to an infant, showing the tracks of infants’ weight growth.

Figure 2 shows the weight growth curves for 519 infants aged from 0 to 12 months old. In order to describe the study explicitly, we divided the weight growth tracks into six equal parts in the picture according to their sequential panel ID. Each curve was corresponding to an infant. Visually, the tracks of infant weight growth are shown to us so that we can assess their nutritional status and screen overweight and obesity timely by comparing with the standard weight growth curves. Table 5 provides the results of relationships between infants sleep and their body weight based on the panel data models. The data was fitted with the pooled effects model, fixed effects model, and random effects model respectively. This study used F test and Hausman-type test to assess the fitting effects of the above models. We got the statistics F = 3.8894, *p* < 0.01, which indicated that fitting the fixed effects model was better than fitting the pooled effects model. The statistics of the Hausman-type test were 388.65, *p* < 0.01, which meant the fixed effects model offered a less biased estimate than random effects. Furthermore, considering the adjusted R-Squared of the three models, the fixed effects model was more suitable for the final analysis. According to the results of diagnostic tests and the fitness of three models, fixed effects method was used to estimate the relationship between sleep and body weight.

The fixed effects model can output 519 intercepts for the 519 infants. Each Infant had a corresponding intercept. Therefore, the final fixed effects model presented as follows:

(4) $${\mathrm{Weight}}_{it}=\alpha_{i}+\mu_{it}-0.049ND_{it}-0.185BF_{it}-0.025NF_{it}-0.113FA_{it}+1.543CF_{it}-0.337SD_{it}-0.246SP_{it}+0.313OA_{it}+0.167TVD_{it},$$

The results of fixed effects model revealed that infants sleep duration were correlated to their body weight and could protect the weight from increasing excessively, after adjusting the infant feeding patterns and socioeconomic factors of their families. However, there was no significant association between sleep problems and infant weight in this study. The present study suggested that variables such as breast feeding, number of breast-feedings per day, and number of formula feedings per day have negative significant relationships with their weight, on the other hand, formula feeding and time of outdoor activities presented positive effects on infant weight. It was found that taking Vitamin D had no statistical difference on infant weight in this study.

## 4. Discussion

Based on the panel data model, this retrospective longitudinal cohort study revealed that insufficient sleep duration in infants was associated with their weight, in contrast, there was no association with between infant sleep problems and their weight, after adjusting the unobserved confounders and feeding patterns.

As described above, extensive studies have been carried out regarding the relationship between sleep and weight among adults, but rarely were there any studies focused on infants. However, excess weight gain during infancy has a profound influence on children and adults, which is worthy of our attention. Previous studies have noted that infant feeding patterns have always been a known factor affecting infant weight, which is consistent with our results [33]. Lower rates of rapid infant weight gain and later obesity have been recognized on breast feeding infants than formula feeding ones [34]. Comparing to the formula-fed infants, this benefit of breastfed ones might be associated with a lower calorie intake, as it was found that the breastfed ones had a better self-regulation in milk intake [35,36]. Similarly, lack of physical activity was a recognized contributor to child obesity. Institute of Medicine guidelines (IOM, 2011) have suggested, in order to prevent overweight or obesity in childhood, infants should be given more daily opportunity to move freely both indoors and outdoors rather than confined to equipment such as baby seats or bouncer seats [9].

After adjusting the feeding patterns and other unobserved confounders, our study found that infant sleep deprivation was a risk factor for infant weight. Tikotzky et al. [22] had indicated a significant correlation between infant sleep duration and obesity after controlling the potential infant’s and family’s confounding factors during the first six months in infancy. Our findings extend their results to one-year-old infants. The beta coefficient of sleep duration for the relative increase in infant weight was −0.337 (kg/h) in our study, which was consistent with that of Tikotzky et al. In addition, previous studies have shown that frequent night waking or settling problems, are related to obesity and body weight [15,16], but no significant difference was observed between them in our study. It might be the result that we did not incorporate data on satisfaction or quality of sleep, which might have affected our results [37]. Therefore, large-scale prospective studies would be required to further explore the association between infant sleep problems with their body weight.

Although the plausible reason for the relationship between sleep duration with the relative increase in infant weight remains unclear, some rational reasons for this could be interpreted. For example, sleep deprivation may cause neurochemicals and appetite changes that are related to the cause of excess weight gain and obesity [13]. Moreover, previous studies have proved that sleep was associated with increased BMI by increasing expression of genetic risks for high body weight [14]. In addition, shorter sleep duration means that the baby could have more opportunities and more time to eat snacks and other kind of foods [38].

One of the main strengths of this study is that we primarily focused on the infants to investigate the relationship between sleep and their body weight, which had been widely examined on adults, but rarely on infants. The study provides more evidence for further studies to demonstrate a correlation of sleep duration and sleep problems with infant weight. Furthermore, the use of community-based panel data was considered as a superior element in this study. Compared with the cross-sectional data, panel data can provide more information by connecting individual experience at different times and screen weight changes timely through weight growth curves. In addition, the time-invariant confounding factors—such as genes and social and parental factors—that may also affect the reliability and validity of parameter estimates could be controlled partly by using the fixed effects model.

However, the research does have limitations. First, the main weakness of the current study is the failure to enlarge the sample size. Mothers of this sample population are highly educated with good salaries at their various working places. Recent study has proved that higher maternal education was associated with higher frequency of exclusive breastfeeding in the first six months, infants and children of better educated mothers more frequently had an adequate dietary diversity. Considering these better feeding behaviors which were good for keeping infant a healthy weight, the effect of infants’ short sleep duration on their body weight might be underestimated [39]. Second, the retrospective design of the current study may present recall bias during the data collecting process [13]. The association between infant sleep and body weight may lead to a bit deviation because of potential inaccuracy of information. Thus, relevant prospective research should be done in the future to overcome this methodological limitation. Third, the measurement of infant sleep duration was based on the mother’s self-report and not by objective actigraph measurement. Previous studies have demonstrated a moderate correlation between self-reported and actigraphy assessed sleep duration and have shown that the mean actigraphy-assessed time spent asleep in the night was 6.74 (SD, 1.02) h, the self-reported sleep duration was more than 1 h longer (7.86 (SD, 1.28) h) [40,41]. Using self-reported sleep duration might introduce some measurement error and result in information bias of association between sleep duration and body weight. Fourth, there are many other relevant factors that are associated with infant weight that were not included in our data analysis because those data were not available. Therefore, future research should consider the multiple potential factors that may be related to infant weight.

## 5. Conclusions

Setting aside the limitations, this study did suggest a reliable relationship between infant sleep duration and infant body weight and provided robust evidence in this field for other studies. Family members, especially parents, should pay more attention to infant sleep duration and help them develop a good sleep habits and ensure adequate sleep duration in order to maintain a healthy weight for infants.

## Figures and Tables

**Table 1:** Baseline characteristics of infants and their mothers in Changsha, China, 2013–2014.

Variable	Sample Size (*n*)	Percentage (%)
Maternal education level		
Junior high or inferior	23	4.4
Senior high	105	20.2
Bachelor’s degree	339	65.3
Master’s degree or advanced	52	10.1
Maternal age		
<25	61	11.8
25–29	194	37.4
30–34	210	40.5
≥35	54	10.3
Per capita income of Chinese households (yuan)		
<5000	280	54.0
5001–<10,000	183	35.3
10,001–<15,000	41	7.9
≥15,000	15	2.9
Marital status		
Single	5	0.9
First marriage	508	97.9
Remarriage	6	1.2
Maternal smoking status during pregnancy		
No	512	98.7
Sometimes	5	0.9
Smoking every day	2	0.4
Paternal smoking status during pregnancy		
No	298	57.4
Sometimes	49	9.4
Smoking every day	171	33.2
Infant gender		
Male	268	51.6
Female	251	48.4

**Table 2:** Nutritional status of 0–12 months old infants among different time points **^1^** (According to longitudinal community-based retrospective birth cohort study conducted in Changsha in 2013–2014).

Nutritional Status	Month Old						χ^2^	Trend	*p*-Value
	0	1	3	6	8	12			
Malnutrition	82 (15.8)	53 (10.2)	27 (5.2)	23 (4.4)	22 (4.2)	27 (5.2)	54.79	Decreasing	<0.001
Normal weight	317 (61.0)	295 (56.8)	265 (51)	268 (51.6)	252 (48.6)	280 (53.9)	10.71	Decreasing	<0.001
Overweight	72 (13.9)	97 (18.7)	102 (19.7)	136 (26.2)	147 (28.3)	141 (27.2)	44.41	Increasing	<0.001
Obesity	48 (9.2)	74 (14.3)	125 (24.0)	92 (17.8)	98 (18.9)	71 (13.7)	4.78	Increasing	0.029

**^1^** Data are presented as number (percentages).

**Table 3:** The characteristics of the subjects in each time point based on panel data **^1^** (According to longitudinal community-based retrospective birth cohort study conducted in Changsha in 2013–2014).

Variable	Months Old					*p*-Value
	1	3	6	8	12	
Number of subjects (*n*)	519	519	519	519	519	
Weight (kg)	4.61 (0.61)	6.77 (1.63)	8.45 (2.32)	9.19 (2.38)	9.97 (1.03)	<0.001
Number of breast-feedings per day (*n*)	9.7 (4.6)	7.7 (4.6)	5.1 (4.7)	3.0 (2.8)	1.3 (2.5)	<0.001
Breast feeding (%)	502 (96.7)	478 (92.1)	413 (79.6)	318 (61.3)	161 (31.0)	<0.001
Number of formula per day (*n*)	1.2 (2.6)	1.5 (2.5)	1.9 (2.3)	2.4 (2.6)	2.6 (2.7)	<0.001
Formula feeding (%)	146 (28.1)	174 (33.5)	269 (51.8)	350 (67.4)	433 (83.4)	<0.001
Complimentary feeding (%)	2 (0.4)	23 (4.3)	483 (93.1)	513 (98.8)	517 (99.6)	<0.001
Sleep problems (%)	18 (3.46)	16 (3.08)	18 (3.46)	16 (3.08)	24 (4.62)	0.344
Sleep duration (hours)	17.78 (1.89)	16.11 (1.67)	14.78 (1.60)	13.67 (1.30)	12.29 (1.90)	<0.001
Time of outdoor activities (hours)	0.16 (0.88)	1.65 (0.72)	2.14 (0.94)	2.45 (0.86)	2.77 (1.34)	<0.001
Taking Vitamin D (*n*)	482 (92.7)	512 (98.7)	507 (97.7)	505 (97.3)	496 (95.6)	<0.001

**^1^** Data are expressed as means (standard deviation) or number (percentages).

**Table 4:** The correlation of infant sleep duration and sleep problems with infant weight across five time points based on panel data (According to longitudinal community-based retrospective birth cohort study conducted in Changsha in 2013–2014).

Variable	Weight for Different Time Points (Months Old)	r/$r_{s}$	*p*-Value
Sleep duration **^1^**	1	−0.100	0.027
	3	−0.229	0.000
	6	−0.297	0.000
	8	−0.064	0.148
	12	−0.010	0.823
Sleep problems **^2^**	1	−0.020	0.644
	3	−0.081	0.065
	6	−0.017	0.703
	8	−0.062	0.162
	12	−0.024	0.594

**^1^** The correlation of sleep duration and infant weight across five time points were examined by using simple correlation; **^2^** The correlation of sleep problems and infant weight across five time points were tested by using Spearman rank correlation.

**Table 5:** Results of relationship between infant sleep and their weight based on the panel data models (According to a longitudinal community-based retrospective birth cohort study conducted in Changsha in 2013–2014).

Variable	Pooled Effects Model			Fixed Effects Model			Random Effects Model		
	B	S.E	*p*-Value	B	S.E	*p*-Value	B	S.E	*p*-Value
Intercept	7.131	0.690	<0.001				8.105	0.880	<0.001
Number of breastfeeding per day (ND)	−0.040	0.007	<0.001	−0.049	0.007	<0.001	−0.040	0.006	<0.001
Breast feeding (BF)	−0.302	0.073	<0.001	−0.185	0.071	0.009	−0.276	0.067	<0.001
Number of formula per day (NF)	−0.038	0.013	<0.001	−0.025	0.013	0.050	−0.031	0.012	0.004
Formula feeding (FA)	−0.094	0.074	0.205	−0.113	0.079	0.150	−0.097	0.072	0.178
complimentary feeding (CF)	1.933	0.068	<0.001	1.543	0.061	<0.001	1.748	0.060	<0.001
Sleep duration (SD)	−0.192	0.013	<0.001	−0.337	0.016	<0.001	−0.258	0.013	<0.001
Sleep problems (SP)	−0.136	0.125	0.278	−0.246	0.168	0.151	−0.197	0.137	0.150
Outdoor activities (OA)	0.330	0.021	<0.001	0.313	0.022	<0.001	0.342	0.020	<0.001
Taking Vitamin D (TVD)	−0.109	0.127	0.389	0.167	0.143	0.260	0.067	0.128	0.601
Birth weight (BW)	0.897	0.057	<0.001				0.898	0.081	<0.001
Gender (GEN)	−0.024	0.046	0602				−0.048	0.065	0.465
Maternal education level (ME)	0.007	0.035	0.844				0.014	0.050	0.779
Per capita income of Chinese households (HI)	−0.063	0.028	0.002				−0.055	0.039	0.161
Gestational age (GA)	−0.045	0.014	0.001				−0.048	0.020	0.017
Maternal smoking status during pregnancy (MS)	0.007	0.115	0.955				0.039	0.164	0.810
Paternal smoking status during pregnancy (PS)	0.032	0.036	0.368				0.032	0.050	0.529
Adjusted *R*^2^	0.73			0.81			0.80		
F statistics	388.81			989.60			575.25		
Overall significance of model	<0.001			<0.001			<0.001		
